# Clinical Features of Essential Tremor in Children

**DOI:** 10.15844/pedneurbriefs-32-2

**Published:** 2018-06-18

**Authors:** Arpita Lakhotia

**Affiliations:** 1 Department of Neurology, Division of Child Neurology, University of Louisville, Louisville, KY

**Keywords:** Essential Tremor, Action Tremor, Movement Disorder

## Abstract

Investigators from Cleveland Clinic Child Neurology Center reported the clinical features of essential tremor (ET) in the pediatric population.

Investigators from Cleveland Clinic Child Neurology Center reported the clinical features of essential tremor (ET) in the pediatric population. A retrospective chart review was performed over 25 years (1984-2011) of pediatric patients diagnosed with ET. Patients meeting core diagnostic criteria of ET and no secondary causes of tremor, and who were less than 21 years of age (with symptom onset less than 18 years of age) were included. 211 children were analyzed of which 61% were male. Mean age of onset was 9.71 years and age at presentation was 14.09 years. Youngest age at presentation was 2 years. Family history was present in 35%. At presentation, 88 patients had progressive course and 95 reported a static course. Functional impairment was present in 55%. 199 patients had bilateral upper extremity tremor at presentation. 12 patients had voice tremor and 20 had tremor at rest. 99 children were available for follow up. Occupational therapy was advised for all patients who had impairment. Pharmacological treatment was given in 30% of patients, of which 75% reported improvement. Within the untreated group, 52% reported unchanged tremor at one year. [1]

COMMENTARY. ET is a common movement disorder in adults and is a progressive disease [2]. Due to the slow progressive nature of the disease, it has a lower degree of functional impairment in childhood, and it becomes more obvious with progression of time. Therefore, it is likely to be underdiagnosed in the pediatric population [3]. Due to these reasons it has not been extensively studied in children, and clinical criteria for diagnosis are extrapolated largely from adult studies.

With 211 patients, this series by Ghosh et al, is the largest series of pediatric ET published by far. This study provides a large pediatric cohort, with clinical characteristics and response to treatment in pediatric ET patients. It brings up an interesting point that some of the secondary clinical criteria for diagnosis of ET may not be applicable to pediatrics, such as duration of symptoms >3 years (as patients themselves may be younger than this age) and response to alcohol. This makes the need to formulate clinical criteria specific to pediatrics furthermore important.

The authors identify clear limitations of the study, with the primary constraint being retrospective nature of data collected. A large number of patients were also lost to follow up which is likely due to lack of significant disability. The diagnosis of ET is clinical, with no biomarker present for diagnosis, which can introduce some level of error with misclassification of patients as ET.

A prospective multicenter study to collect clinical characteristics of pediatric patients with ET would help further corroborate data obtained from this study. This will be helpful for formulating pediatric specific guidelines and diagnostic criteria for ET. Controlled trials for pharmacotherapy in pediatric ET are also required. In the current age of personal genomics, identifying cohorts of families with ET would help decoding the underlying genetic changes in ET.

## Disclosures

The author has declared that no competing interests exist.
